# Drug-Dosing Adjustment in Dogs and Cats with Chronic Kidney Disease

**DOI:** 10.3390/ani12030262

**Published:** 2022-01-21

**Authors:** Francesca De Santis, Andrea Boari, Francesco Dondi, Paolo Emidio Crisi

**Affiliations:** 1 Faculty of Veterinary Medicine, Veterinary Teaching Hospital, University of Teramo, Località Piano d’Accio, 64100 Teramo, Italy; fdesantis@unite.it (F.D.S.); aboari@unite.it (A.B.); pecrisi@unite.it (P.E.C.); 2 Department of Veterinary Medical Sciences, Alma Mater Studiorum, University of Bologna, Via Tolara di Sopra 50, 40064 Bologna, Italy

**Keywords:** chronic kidney disease, small animals, dose adjustment, nephrology, pharmacology

## Abstract

**Simple Summary:**

Adjusting drug dosages in dogs and cats with chronic kidney disease (CKD) can be challenging in clinical practice due to the lack of specific indications in the current literature; moreover, the evaluation of renal function through the measurement of glomerular filtration rate (GFR), which is unanimously considered as a requisite for most adjustment strategies, is often hard to perform in clinical settings. Therefore, the present review aims to provide practical guidelines for dosage adjustment in CKD patients through an overview of the available literature.

**Abstract:**

Chronic kidney disease is a common kidney disorder in adult and aged dogs and cats; the management of associated complications and comorbidities generally requires a life-long medical treatment to ensure a good quality of life of affected patients. However, indications and the literature on drug dosing in dogs and cats with chronic kidney disease are often lacking. The aim of this review is to revise the current literature on drug dosing in canine and feline patients with renal impairment, with a special focus on the most commonly used medications to manage chronic kidney disease and possible comorbidities.

## 1. Introduction: Chronic Kidney Disease and Implications in Drug Dosing

Chronic kidney disease (CKD) is the most commonly recognized form of kidney disorder in dogs and cats and, in most instances, it represents an irreversible and progressive condition [1]. The prevalence of CKD has been estimated to be 0.5–1.0% in dogs and 1.0–3.0% in cats, with a higher prevalence in older animals and a rate as high as 80% in the geriatric cat population [2,3,4].

The clinical consequences of CKD reflect the extent of a reduction in renal function rather than the impact of structural lesions [3]. Given the progressive nature of the disease, these patients often require lifelong medical treatment to limit the progression of renal injury and improve their quality of life. The conservative medical management of CKD consists of a supportive and symptomatic therapy designed to ameliorate clinical signs of uremia, correct fluid and electrolyte imbalance, provide adequate nutritional requirements and slower the progression of renal failure [5]. Antihypertensive and antiproteinuric drugs, phosphate binders, antiemetic and antacid medications are usually administered to manage the clinical signs and long-term complications related to renal failure [5,6,7]. Furthermore, in dogs with protein losing nephropathy (PLN), which are considered at risk for the development of thrombosis, antithrombotic prophylaxis is recommended [8].

Liver metabolism and renal excretion are the major drug-elimination pathways [9]. For this reason, patients with an impaired renal function could require adjustment of the usual drug-dosage regimen, not only to overcome serious adverse reactions because of excessive drug accumulation but also to avoid treatment failure [10,11]. In fact, either sub- or supratherapeutic dosing can occur when appropriate dose adjustments are not made in patients with kidney disease, and both have negative effects on patient outcomes [10].

In veterinary clinical practice, dose adjustment for CKD patients is rarely performed: there is no widely available, easy-to-perform method to measure the glomerular filtration rate (GFR), which is generally required to establish an appropriate dosing strategy. Furthermore, there are no practical guidelines to perform dose adjustments in animals with CKD. Indeed, information on specific dose adjustment in dogs with renal impairment is sparse [12] and the available information for cats is often extrapolated from canine or human reports [13].

The present review describes the mechanisms of altered drug handling in CKD and discusses the rationale of dose adjustment regimens in the most commonly used classes of medication in CKD management and related complications; moreover, the effective need to adjust the dose of other commonly used drugs in clinical practice, and their potential impact on renal function, will be analyzed.

## 2. Evaluation of Renal Function

A thorough assessment of renal function is required to establish an appropriate dose adjustment. Glomerular function is generally considered the best indicator of renal function and can be evaluated by means of both indirect and direct tests.

The measurement of GFR is deemed the most sensitive test directly measuring kidney filtration and excretion that can detect decreases in organ function [14,15]. An evaluation of GFR may provide critical information on kidney function far earlier than the more widely used markers of renal function in dogs and cats with unexplained polyuria and polydipsia and might help predict the risk of developing overt renal failure after nephrectomy in animals with unilateral kidney disease [16]. Furthermore, the measurement of GFR could be useful for the diagnosis of CKD before the onset of azotemia, for ruling out pre-azotemic acute kidney injury (AKI) and for dose adjustments of chemotherapy and other potentially nephrotoxic medications [16,17].

Glomerular filtration rate is defined as the volume of ultrafiltrate produced by glomerular filtration per unit of time and can be measured using the urinary or plasmatic clearance of appropriate markers, where the clearance describes the relationship between the transfer rate of the marker and its concentration in urine or plasma, respectively [14,15]. Thus, an ideal GFR marker should be freely filtered by the glomeruli, not reabsorbed or secreted by the renal tubule, nor metabolized or produced by the kidney, and should not alter the GFR value itself; when the plasma clearance approach is used, the marker should also be totally cleared by the kidneys [14]. The markers that are currently used to measure GFR in animals include inulin, exogenous creatinine, radionucleotides and iohexol. Due to its ease of use, low cost and wider availability, plasma clearance of iohexol is the most commonly used marker of GFR in veterinary and human medicine [17,18]. The principle underlying plasma clearance methods is the repeated measurement of the elected marker in several blood samples taken over time, following the initial administration. Clearance (Cl) is expressed as mL/min/kg and calculated using the formula: Cl = dose/AUC, where AUC is the area under the plasma disappearance curve of the marker [19]. Sampling should ideally continue until all the administered marker is excreted. The longer the period of collection, the more accurate the GFR measurement. However, given that repeated blood sampling is not always feasible in clinical practice, correction formulae derived from human medicine (e.g., the Brøchner–Mortensen formula) have been proposed and applied in dogs and cats to estimate GFR from the clearance, determined with a limited sampling strategy [17,18,20,21,22].

As with indirect makers of renal function, GFR is also influenced by several extra-renal factors, including sex, age, breed, dietary protein intake, hydration status, sodium balance, and day-to-day circadian rhythm [19]. Furthermore, given the high variability that exists in dogs and cats, a standardization of GFR to body size is needed. To this end, GFR has been related to body surface area, body mass and extracellular fluid volume; in veterinary medicine, GFR is generally related to body mass and is thus expressed in mL/kg/min [14,19]. Most published normal values for GFR are between 2 and 5 mL/kg/min in dogs and cats and a value below 1.5 mL/kg/min is generally assumed to be abnormal in average-sized dogs [23]. A cut-off value of 2.1 mL/kg/min should be considered for a body weight of <10 kg and about 1.2 mL/kg/min for dogs with a body weight of >45 kg [15]. Plasma clearance references were proposed for cats, considering a range of 1.0–3.5 mL/kg/min for iohexol and 1.3–3.8 mL/kg/min for creatinine [22]. To date, despite its accuracy in evaluating renal function in comparison with indirect markers, the assessment of GFR is rarely performed in veterinary clinical practice due to its poor feasibility and limitations in the interpretation of results.

Among the indirect methods used to assess renal function, the combination of plasma or serum creatinine (sCr), urea concentrations and urine specific gravity (USG) is commonly evaluated in veterinary medicine [24]. Based on these variables, the International Renal Interest Society (IRIS) has proposed a scoring system for staging CKD in dogs and cats to assist clinicians in determining the appropriate therapy, forecasting prognosis, and implementing follow-up steps required for appropriate disease management. According to the IRIS staging guidelines, renal function has to be measured in a hydrated and stable patient, by fasting sCr concentration (possibly combined with symmetric dimethylarginine, SDMA), on at least two occasions. The subsequent sub-classification is then based on the evaluation of proteinuria and blood pressure [25].

Serum creatinine concentration is currently considered the best indirect marker of GFR. Creatinine is produced by the degradation of creatine and creatine phosphate in skeletal muscles at a relatively constant rate and, once in the blood stream, it is completely filtered by the glomeruli, without significant tubular reabsorption or secretion [14,15,26]. The increase in sCr concentration above the reference interval is assumed to be representative of a loss of function of at least 67–75% of nephrons [15]. The relationship between sCr concentration and GFR is represented by a steep curvilinear trend, which means that a significant reduction in GFR is required before a corresponding increase in sCr concentration is seen. Nevertheless, even sCr values within the reference interval can be used to predict a decline in GFR over time, provided that the non-renal factors influencing creatinine remain stable [24,27,28]. Serum creatinine concentration can, therefore, be considered a more sensitive marker in the late course of CKD rather than in the early stages. Although a high sCr concentration is typically associated with severe renal dysfunction, some other conditions should be considered, such as the high muscle production of creatinine, reduction in extracellular fluid volume and intestinal absorption of exogenous creatinine. Hence, physiological factors such as age, muscle mass, breed, exercise and the effect of meals should be taken into account when an increase in plasma creatinine is observed. Furthermore, sCr has shown lower sensitivity as a marker of renal function in dogs with portosystemic shunts and in cats with hyperthyroidism [15,29]. To overcome the aforementioned limits of sensitivity and sensibility, the IRIS staging guidelines recommend that sCr should be evaluated in fasted, stable, well-hydrated patients on at least two occasions. Moreover, the result should be interpreted considering the breed, muscle mass and age of the animal [24].

Urea is the main form in which nitrogen is eliminated from the body and its concentration in serum or plasma can be expressed as blood urea nitrogen (BUN) [15]. Serum urea concentration is increased in renal failure; however, in contrast to creatinine, it is considered a worse indirect indicator of GFR due to its tubular re-absorption. Moreover, BUN can be increased in several non-renal conditions such as prerenal azotemia, high-protein diet, gastrointestinal bleeding, tetracycline or corticosteroid administration. Conversely, its concentration can be decreased in hepatic insufficiency, polyuria and polydipsia and low-protein diets. Given its poor sensibility and specificity as marker of renal function, it is generally recommended that BUN is evaluated in conjunction with creatinine concentration and USG measurements [30].

Urine-specific gravity provides important information about the renal urine concentrating capacity and it is widely reported that, in dogs with pre-renal azotemia, USG should exceed 1.030, whilst in cats, it should exceed 1.035 [15,31]. However, cats may also maintain some urine concentration ability, despite significant reductions in renal mass, especially when dehydrated [24,32].

Symmetrical dimethylarginine is a compound produced in cells through the methylation of arginine residues and it is subsequently released into the circulation during proteolysis [33,34]. In recent years, research has focused on how SDMA may be utilized in veterinary medicine, and assays for its measurement are currently available in clinical practice. Despite analytical and intra-individual variability, there is evidence that SDMA is not influenced by muscle mass, and is thus likely to provide a more accurate representation of GFR than creatinine, especially in patients with sarcopenia [35,36]. Based on this concept, SDMA has been incorporated into the IRIS staging system. The relationship between SDMA and serum or plasma creatinine and GFR seems to be very similar, but SDMA can detect a decline in renal function earlier, highlighting its possible role as an earlier marker of CKD [24,28,36,37]. Nevertheless, the possible influence of non-renal diseases on the specificity and sensibility of SDMA as a marker of renal function is still under investigation.

## 3. Mechanisms Underlying Altered Renal Drug Handling in CKD

The mechanisms responsible for the altered renal drug handling in renal impairment are different and complex, and involve both pharmacokinetic and pharmacodynamic processes [38].

Pharmacokinetics is a branch of pharmacology that describes the body’s effect on a drug, reflecting the physiologic processes of absorption, distribution, metabolism and excretion; all of these phases may be altered in patients with kidney disease. Conversely, pharmacodynamics studies the effects of the drug on the body, including interactions between the drug, its target and downstream biochemical effects [10].

The adsorption of drugs from the gastrointestinal tract may be highly variable in CKD patients, and can increase due to an impairment in the gut wall barrier function or due to an abnormal function or expression of efflux transporters [39]. Furthermore, the interactions between several orally administered drugs should be considered, as in the case of phosphate binders that significantly influence the intestinal absorption of other orally administered drugs [40].

An expansion in the volume of distribution (Vd) is reported in advanced CKD because of fluid retention, hypoalbuminemia and the subsequent decreased protein binding [11,39]. Specifically, the reduction in plasma protein binding is a consequence of several mechanisms, such as hypoalbuminemia, that occur in protein-losing nephropathies (PLN), the accumulation of endogenous substances, which competitively displace acidic drugs from the binding sites of albumin, or even conformational changes in the binding sites. In fact, uremic toxins such as urea, indoxyl sulphate and cytokines may be responsible for transcriptional, translational or post-translational modifications to several transporter and enzyme functions [41,42,43,44].

Glomerular filtration, tubular secretion and tubular reabsorption are the three main renal drug excretion pathways, although other pathways, such as metabolism and P-glicoprotein activity, can contribute [9]. The glomerular filtration of a drug is a passive process involving the plasmatic unbound drug fraction, and is dependent on the renal blood flow available for filtration. The tubular secretion is an active system of transport that occurs via membrane transporters, such as organic cation transporters (OCT) and organic anion transporters (OAT) located in the proximal tubule of the nephron; competition for the transporters can be responsible for drug interactions. The tubular reabsorption is mainly a passive transport process allowing low-molecular-weight, lipophilic and un-ionized molecules to be passively reabsorbed based on diffusion principles. Less commonly, the reabsorption process in the proximal tubule can be actively mediated by specific transporters [9].

The renal excretion of drugs in CKD is generally decreased as a result of the reduction in GFR. Nevertheless, the mechanisms underlying drug excretion in renal impairment are complex. The practical approach based on the “intact nephron hypothesis”, which assumes that renal drug clearance will almost entirely decrease in proportion to the reduction in GFR, is subjected to several limits and is not suitable for all drugs, types of kidney disease and different GFRs [45,46]. In fact, recent evidence suggests that renal drug handling pathways may not decline in parallel, and thus the evaluation of each of these pathways may be more adequate [9,10,39].

Only a few drugs are eliminated almost entirely unchanged by the kidneys, and most of them undergo metabolization reactions [11]. Although the renal metabolism is generally lower than that of the liver or gastrointestinal tract, it has been suggested that the kidneys may be responsible for 15–40% of the metabolization of some drugs, mainly through the reaction of sulphation and glucuronidation [9,47]. In patients with kidney dysfunction, the renal excretion of parent drugs and/or active metabolites is often impaired, possibly due to alterations in drug-metabolizing enzyme activity caused by the accumulation of uremic toxins, leading to their excessive accumulation in the body [48,49]. Moreover, the non-renal clearance of drugs in patients with CKD can be additionally compromised due to reduced metabolic enzyme activity and alterations in the function of transporters caused by uremic toxins [11].

## 4. Rational Design of Dosing Regimens in CKD Patients

The goal of rational drug dosing is to maximize the benefits while minimizing the risks related to a given medication. Although indications of dosage adjustment in patients with kidney disease are often provided by the product label, several limitations should be considered; for example, the difficulty of testing pharmacokinetics in a large sick population, in which interpatient pharmacokinetic variability exists. Moreover, this is further complicated when a heterogeneous mix of etiologies is implied. Moreover, a dosage adjustment is generally based on the simplistic assumption that only renal clearance is changed. As a result, manufacturers may recommend that a given medication is contraindicated in patients with advanced kidney disease [39].

A common practical approach for drug dosing in patients with renal disease is to use a conservative approach with progressive dose escalation; however, this method may be reasonable for drugs that yield a clinical benefit from long-term treatment but is not indicated for drugs requiring a rapid onset of effect, such as antibiotics or immunosuppressive medications [39].

The main principles that should be taken into account when a dose adjustment is considered are the therapeutic target, the initial dose, the maintenance dose and the frequency of administration [39]. The dose fraction (Kf) represents a key factor for dosages adjustment. It is defined as Kf = GFRr/GFRn, where GFRr and GFRn are the GFR values in renal impairment and healthy conditions, respectively [50].

The “constant-interval, dose reduction” method is one of the possible applicable strategies for dose adjustment and is more indicated when the dosage interval is less than the elimination half-life, and with drugs with a narrow therapeutic index, such as aminoglycosides; this method is generally associated with the achievement of a more constant drug concentration [51]. The dosing interval is unchanged and the dose is multiplied by Kf [50,52]; alternatively, a dose reduction to 75% and 50% of the standard dose could generally be expected for moderate and severe renal disease, respectively [53].

In the “constant-dose, interval extension” method, the dose is unchanged and the dosing interval is increased by dividing it by Kf. From a practical perspective, a formula based on serum creatinine can alternatively be used to determine the dose interval, as follows: new interval = normal dosing interval × (patient creatinine/normal creatinine) [54]. However, given that the change in GFR is considered the best indicator of renal dysfunction and alterations in drug renal clearance, the first step in dosage regimen adjustment should be the determination of GFR value in the patient [15,55,56]. This method works well with medications that have a broad therapeutic window and a long half-life, such as penicillin and cephalosporins [51]. It is worth noting that, for antibacterial drugs with a concentration-dependent kill rate, this method is appropriate, while for those with time-dependent activity, the “constant-interval, dose-reduction” method is recommended to maintain plasma concentrations above the minimum inhibitory concentration (MIC) [50,51,52,57]. When a dosage adjustment is required, the risks of subtherapeutic serum concentrations must be weighed against the risks of adverse events. Consequently, combining dose reduction with interval extension could be a valuable option in the CKD population [51].

Provided that the time required to reach a steady state is generally increased in renal impairment, the administration of a loading dose at the beginning of treatment could be an effective strategy to allow therapeutic concentrations to be reached more rapidly [52]. This concept should be taken into account for antimicrobial drugs, for which a loading dose is often required to bring the serum level above MIC within a reasonable time period [51]. The appropriate loading dose could be ascertained using the following calculation: loading dose = Vd × IBW × Cp, where Vd is the volume of distribution measured in liters per kilogram, IBW is the ideal body weight in kilograms, and Cp is the desired medication plasma concentration in milligrams per liter [51]. Alternatively, if the constant-interval method is employed, the usual dose can be administered as a loading dose, followed by the calculated reduced dose. If the constant-dose method is used, the initial two doses should be given according to the usual interval [43].

Therapeutic drug monitoring (TDM) consists of monitoring plasma drug concentrations at regular intervals to optimize the effectiveness of the treatment. If drugs with a narrow therapeutic index are used under renal impairment conditions, this should be performed if renal dysfunction progresses, or adverse reactions or therapeutic failure occur [50,55,58].

In the following sections, an overview of the main literature on drug-dosing in CKD canine and feline patients will be revised, and recommendations for dose adjustments to the most commonly used drugs will be provided. A summary of the main indications and recommendations is provided in Table 1.

## 5. Antimicrobial Agents

Antimicrobials are frequently prescribed in CKD patients due to the high prevalence of urinary tract infection (UTI), which is reported to be approximately 15–30% in cats [115,116] and 8% in dogs with CKD [117].

Renal excretion is the major route of elimination for almost all antimicrobials, and dosage adjustments are commonly made in human patients with renal insufficiency due to concerns regarding their potential nephrotoxicity and efficacy [118]. The major variables for bactericidal drugs are the peak of concentration and the area under the plasma concentration time curve, while the “constant-interval, dose reduction method” seems to be more appropriate to maintain therapeutic levels of bacteriostatic antimicrobials [55].

Varying degrees of nephrotoxicity are reported for aminoglycosides, cephalosporins, tetracyclines and sulfonamides; a negligible nephrotoxic potential is attributed to penicillins and fluoroquinolones due to their high therapeutic index and partial renal excretion, respectively [55].

### 5.1. Aminoglycosides

Aminoglycosides are among the best choices for the treatment of severe Gram-negative aerobe bacterial infections [59,119,120]. The introduction of broad-spectrum antibiotics with better safety profiles, such as fluoroquinolones and extended-spectrum β-lactam antimicrobials, has led to a reappraisal of the indications for aminoglycoside therapy. To date, their use is limited to the short-term treatment of infections caused by susceptible microorganisms that are resistant to less toxic antibiotics [59].

Aminoglycosides are poorly adsorbed from the gastrointestinal tract and have a low protein binding. These drugs are almost entirely eliminated by glomerular filtration; however, they progressively accumulate in the renal cortex, causing their toxic effect [55]. Among other side effects, the nephrotoxic risk is a major concern, especially when parenterally administered, for long-term therapies and when pre-existing renal impairment conditions are present. In addition, the risk associated with topical administration should not be underestimated [55,121]. The risk of nephrotoxicity increases with the treatment duration, as aminoglycoside nephrotoxicity is cumulative [59,122]. Neomycin and gentamicin are the most nephrotoxic aminoglycosides, while tobramycin is less nephrotoxic than gentamicin at equivalent doses [55]. The nephrotoxic effect, which is due to acute tubular necrosis, is usually reversible with early discontinuation, and this risk may be reduced by using short-term therapies and allowing the plasma drug concentration to drop below the recommended concentrations doses. Nevertheless, caution should be used for all aminoglycosides in patients with preexisting renal disease and conditions such as dehydration, fever or sepsis [55,59]. Although cats are reported to be less susceptible to gentamicin-induced nephrotoxicity than dogs, its administration in both dogs and cats affected by CKD is discouraged [7].

For many years, aminoglycosides were administered in multiple daily doses. However, the total daily dose in one administration lowers the risk of toxicity, as it reduces the proportion of the total daily dose that can accumulate within the kidney [59]. Indeed, many in vitro and in vivo studies suggest that administration in a single daily-dosing (SDD) regimen is as effective as, or more effective than, conventional regimens and reduces the associated toxicity [123,124,125,126]. Aminoglycosides provide a post-antibiotic effect (PAE), which is defined as the ability to promote a bacteriostatic effect even after serum drug concentrations have fallen below the minimum MIC of the organism in question. This peculiarity allows for longer dosing intervals, according to the fixed-dose interval extension method [55,59,120,125,127].

Due to the interpatient variability in pharmacokinetic variables, such as volume of distribution and renal function, TDM has been the standard of care in people treated with traditional multiple-daily-dosing regimens to ensure adequate peak concentrations and prevent toxicity; moreover, TDM is also recommended with SDD when treatment duration is longer than 5–7 days or for patients at high risk of toxicity [59,120,125]. As toxicity is both dose- and time-dependent, the treatment duration should be minimized as much as possible. 

The daily monitoring of renal function through the evaluation of increases in plasma creatinine, urine sediment for granular casts and reduction in urine output is recommended; if any sign of toxicosis is observed, the administration should be interrupted [59,128].

Recommended gentamicin doses under a SDD regimen via intravenous (IV), intramuscular (IM) or subcutaneous (SC) routes ranges between 9 and 14 mg/kg in dogs and 5 and 8 mg/kg in cats [129]. Serum peak concentrations 30 min after IV administration or 60 min after IM administration could be evaluated in TDM and should fall between 15 and 20 mg/mL; alternatively, a concentration measured 2–4 h before the next dose should not exceed 1 µg/mL or be undetectable [59]. Recommended doses for amikacin at a SDD regimen range between 15 and 30 mg/kg in dogs and 10 and 14 mg/kg in cats [129]. Serum peak concentrations should fall between 30 and 40 mcg/mL; through concentration should not exceed 2.5 µg/mL or be undetectable [59].

Association with other nephrotoxic or nephroactive agents, such as other aminoglycosides, cephalosporins, nonsteroidal anti-inflammatory drugs (NSAID, phenylbutazone), cisplatin, cyclosporine or diuretics, should be avoided [55,59,60].

### 5.2. Penicillins

Penicillins exert a bactericidal action against Gram-positive and some Gram-negative bacteria. However, although the nephrotoxic effect in dogs and cats attributed to penicillin is generally negligible because of the high therapeutic index of these antibiotics [55,60,121], it is worth to consider the potential impact on kidney function in patients with renal impairment.

In human patients with decreased renal function, potentiated penicillin has a decreased renal clearance, resulting in higher serum concentrations in comparison with patients with normal renal function; thus, since potentiated penicillin is predominately excreted by the kidneys, dose adjustment of potentiated penicillin (i.e., decreased dose or decreased frequency) has been recommended in human patients with a severe reduction in GFR (i.e., <30 mL/min) to avoid excessive drug accumulation [57,130].

Similarly to those in humans, penicillin-related side effects seem to be limited in dogs with renal insufficiency due to their high therapeutic index. However, since it has been shown for ampicillin that drug accumulation might occur beyond 48 h of administration, dose adjustment should be considered [55,61]. In particular, in dogs with severe azotemia (creatinine > 4 mg/dL), after a single 22 mg/kg ampicillin dose, a slower clearance and increased plasma concentrations of ampicillin has been reported; in such cases, an interval extension can be adopted in order to reduce the risk of drug accumulation while still maintaining an effective therapeutic level for susceptible bacteria [61].

The dose adjustment of penicillin in cats with impaired renal function appears to be unnecessary due to the wide therapeutic window of these antimicrobials, which could be classified as “probably safe” in CKD patients [7,13]. The side effects of amoxicillin–clavulanic acid were evaluated in feline patients with and without azotemic CKD, with altered pharmacokinetics observed in cats with azotemic CKD; however, the need for dose adjustments in azotemic feline patients remains to be determined [62].

### 5.3. Cephalosporins

Cephalosporins are broad-spectrum bactericidal agents, active against Gram-positive and Gram-negative bacteria. They are classified into four generations, characterized by an increasing resistance to β-lactamase destruction, and often by an extended, although variable, *spectrum* [60].

The pharmacokinetic properties of cephalosporins are similar to those of penicillin. The plasma protein binding is <40% and the main route of elimination is tubular excretion, although cephalothin, cephapirin and cefotaxime have a substantial hepatic metabolism [131].

Cephalosporins are a class of relatively safe antimicrobial agents; nevertheless, high doses or very prolonged use have been associated with interstitial nephritis and tubular necrosis [57,60]. With the exception of those which are mainly eliminated by the extrarenal route, such as cefoperazone or cefixime [118,132], cephalosporins clearance is decreased in human patients with renal failure, and a dosage regimen adjustment is recommended if a moderate-to-severe reduction in GFR occurs [63]. These antimicrobials are suitable for interval adjustment; for instance, the standard q8h interval of cefazolin can be adjusted to q12h in patients with CKD Stage 3. In very severe cases (i.e., serum creatinine > 8.5 mg/dL), the dose interval should be extended at 48 h [64]. Once-daily dosing is recommended for cefotetan at IRIS Stage 3 and administration every other day (EOD) can be recommended at IRIS Stage 4 [64]. Similarly, the risk of nephrotoxic effects should be considered in renal-impaired dogs, especially when associated with aminoglycosides [55,60]. Dose adjustment in cats is recommended in moderate or severe CKD (IRIS stages 3 and 4) [7].

### 5.4. Sulfonamides

Sulfonamides are bacteriostatic agents for Gram-positive and several species of Gram-negative bacteria. They can be highly bound to serum proteins with possible competitive interactions with other drugs, but the specific extent of binding is species- and drug-dependent. Sulfonamides are excreted via glomerular filtration in their unbound form and through tubular secretion. Metabolism is principally performed by the liver, through acetylation and glucuronidation reactions, but the extra-hepatic metabolism is also involved [60].

Trimethoprim-sulfonamide is a potentiated antimicrobial agent that, in contrast with sulfonamides alone, provides a bactericidal action. The association of trimethoprim with different sulfonamides has been labeled, such as sulfadiazine and sulfamethoxazole. There is considerable controversy regarding the dosage recommendation and frequency of administration of these combinations, due to inconsistencies in the pharmacokinetic data. Furthermore, since different organisms have different MIC values and the optimal ratio of trimethoprim-sulfa also differs from organism to organism, this problem is exacerbated [60,85].

Sulfonamides may induce a variety of adverse effects. Specifically, the possible onset of crystalluria, as a result of the sulfonamide’s precipitation in renal tubules, should be taken into account due to its potential impact on kidney function; thus, although a tubular obstruction has been anecdotally reported in animals receiving sulfonamides, dehydration should be avoided [65]. Since the acetylated metabolites are characterized by a lower solubility, crystallization in the urine can occur with some sulfonamides, particularly in an acidic environment. Prolonged administration and highly concentrated urine may also be contributing factors in the onset of crystalluria, hematuria and renal tubule obstruction [60,85]. In dogs, acetylation reactions do not occur and tubular secretion does not appear to be a major route of elimination, making this species less susceptible to renal tubular damage [55]. Nevertheless, because of the risk of accumulation, they are contraindicated in patients with severe renal impairment (i.e., serum creatine > 8.5 mg/dL) and should also be used with caution in patients with diminished renal function or urinary obstruction. It has been recommended that the normal dose should be halved in dogs at IRIS Stage 4, while no adjustments have been suggested for plasmatic creatinine < 5.0 mg/dL [12,64].

Furthermore, delayed hypersensitivity reactions caused by sulfadiazine, sulfadimethoxine or sulfamethoxazole have primarily been described in dogs [133,134], with a suspected increased susceptibility in Doberman Pinschers [135,136,137]. Glomerulopathy, polymyositis, polyarthritis, dermatological signs, fever, hepatotoxicity and hematological disorders have been encountered as manifestations of hypersensitivity reactions [65]. Acute interstitial nephritis is also reported in isolated case reports [138]. For both sulfonamides alone and the association trimethoprim-sulfonamides, a 50% dose reduction is recommended in cats with moderate or severe CKD [7,64].

### 5.5. Tetracyclines

Tetracyclines are broad-spectrum bacteriostatic antibiotics. With the exception of doxycycline and minocycline, they are generally excreted unchanged in urine and, to a lesser extent, in bile. It has been shown for tetracycline and oxytetracycline that renal impairment in dogs induces a dramatic decrease in body clearance and the apparent volume of distribution, as well as a prolonged half-life and drug accumulation with repeated dosing [55,60,85]. In advanced CKD, with the exception of doxycycline, the accumulation of tetracyclines and their metabolites can increase the risk of adverse effects [7,55]. Therefore, when indicated, doxycycline has been identified as the tetracycline of choice in these patients because of its lower rate of renal elimination. Minocycline can also be used in patients with moderate renal insufficiency without dosage adjustment, which is required for oliguric renal failure [60].

### 5.6. Fluoroquinolones

Fluoroquinolones exert bactericidal activity primary against most Gram-negative bacteria, *Mycoplasma* and some Gram-positive bacteria [55].

Fluoroquinolones undergo renal and non-renal elimination pathways; approximately 15–50% are eliminated unchanged by glomerular filtration and tubular secretion, while a significant proportion is metabolized by both the liver and kidneys and eliminated in feces and urine as a few active metabolites. Approximately 10–40% of circulating enrofloxacin is metabolized to ciprofloxacin in most species, including dogs and cats [55,66]. Due to the dual means of elimination, patients with severely impaired renal function may have slightly prolonged half-lives and higher serum levels, which may not require dosage adjustment [55,139]. In CKD feline patients with moderate disease, an interval extension is recommended because of the high risk of retinopathy at standard therapeutic doses, especially with enrofloxacin [7]; alternatively, less retinotoxic fluoroquinolones, such as marbofloxacin, should be considered [64].

## 6. Anti-Inflammatory Drugs

### 6.1. Non-Steroidal Anti-Inflammatory Drugs

Non-steroidal anti-inflammatory drugs (NSAIDs) are largely used in small-animal practice for their anti-inflammatory, antipyretic and analgesic properties [55,67,140,141]. Nevertheless, NSAIDs also have some undesirable effects that can be seen in both therapeutic dose and overdose situations, although the overall incidence of adverse effects in companion animals is not known [67,68,142]. The most commonly reported adverse effects of NSAIDs are gastrointestinal, renal, hepatic, and coagulation disorders [143,144]. NSAIDs are regarded as harmful for patients with CKD and clinical guidelines for humans currently recommend the avoidance of prolonged NSAID use in CKD with GFR > 30 mL/min/1.73 m^2^ and complete avoidance with GFR < 30 mL/min/1.73 m^2^ [145,146,147]. NSAID-associated nephrotoxicity is predominantly hemodynamically mediated, resulting in a reversible reduction in renal blood flow (RBF) and/or GFR and in severe cases, acute ischemic tubular injury that may lead to acute kidney injury (AKI) [70,147,148]. In dogs with CKD, RBF maintenance is increasingly dependent on prostaglandins; therefore, the use of NSAIDs increases the risk of renal vasoconstriction thorough a decreased prostaglandins production, meaning that AKI from NSAIDs is more likely to occur in dogs that already have decreased renal function [70].

In order to reduce the incidence and the severity of these complications, several molecules with better safety profiles have been developed over the years. The primary mode of action of NSAIDs is blocking the cellular expression of the enzyme cyclooxygenase (COX) in cell membranes, resulting in diminished prostaglandins (PGs) and thromboxane production. COX-2 selectivity and the sparing of COX-1 were reported as safety factors to reduce NSAIDs… [143]. However, besides its proinflammatory effect, COX-2 has a secondary but important role in the regulation of certain physiological functions, whose inhibition would likely lead to adverse consequences [140]. While COX-2 selectivity seems to be clinically significant in reducing gastrointestinal (GI) adverse reactions, it may have little effect on potential renal toxicity, liver toxicity or coagulative functions. After the GI effects, nephrotoxicity is the second most important NSAIDs safety concern and, since COX-1 and COX-2 are both constitutively present in normal kidneys, seems unrelated to COX selectivity [143,149].

Nonsteroidal anti-inflammatory drugs might negatively affect renal function through the suppression of homeostatic renal PGs; this inhibition is particularly important under hypovolemia and hypotension conditions, such as after trauma or in the perioperative period, due to the risk of developing ischemic injury [143,144].

Non-steroidal anti-inflammatory drugs are generally well-absorbed by the gastrointestinal tract, they are highly bound to plasma proteins and have a low apparent volume of distribution. They undergo hepatic transformation and the excretion of unchanged drug by the kidney is usually low with profound interspecies differences in the elimination half-lives that exist among molecules. In neonates and patients with renal or hepatic disease, the half-lives of NSAIDs are usually increased [55,67]. Non-steroidal, anti-inflammatory drugs are contraindicated in patients with renal insufficiency, dehydration and hypotension, and the concurrent use of other potentially nephrotoxic drugs, such as aminoglycosides, is discouraged [140,143]. Screening and monitoring are essential parts of the judicious use of NSAIDs to identify preexisting pathologic conditions or early signs of adverse effects, especially in older animals and when long-term use is expected [143,150].

Carprofen, deracoxib, firocoxib, grapiprant, meloxicam and robenacoxib are some of the approved molecules for the management of osteoarthritis, perioperative pain and inflammation in dogs; these molecules have all been approved for long-term use, with the exception of robenacoxib. To date, only meloxicam and robenacoxib have been approved in cats for the short-term management of perioperative pain and inflammation [69]. Although the cautious use of NSAIDs is usually recommended in cats due to the limited hepatic glucuronidation, there is a significant body of evidence highlighting their value and safety in this species, particularly concerning newer compounds. While molecules that are mainly metabolized by glucuronidation, such as aspirin, acetaminophen, and carprofen, have relatively prolonged elimination half-lives in cats compared with dogs, drugs cleared by oxidative enzymes, such as piroxicam and meloxicam, have been shown to have similar or reduced half-lives in cats [144]. Furthermore, other NSAIDs elimination pathways have been identified in cats, such as the elimination of unchanged flunixin through organic anion transporters into bile [151], and thioesterification of ketoprofen [152].

Data regarding the nephrotoxicity of NSAIDs in cats or the use of these drugs in feline patients with preexisting renal disease are controversial [153]; however, from the nephrological perspective, all these drugs should be used with extreme caution in renal disease. Despite anecdotal reports of acute renal failure and death associated with the use of meloxicam in cats [67], the short-term administration of NSAIDs seems to be an uncommon cause of nephrotoxicity [154]. Moreover, long-term NSAIDs therapy was found to be safe and effective when administered to cats with osteoarthritis for approximately 6 months [155]. Recent publications suggest that NSAIDs can also be administered to cats with CKD, providing a high level of monitoring and care [156,157,158,159]. Furthermore, recent evidence suggests that the use of NSAIDs in cats with CKD may lead to a slowdown in the progression of the disease, probably due to the direct renal anti-inflammatory effect or to the relief of pain and a subsequent appropriate food and water intake [156]. Nevertheless, a recent publication found that, although low-dose meloxicam administration did not negatively affect the serum creatinine concentration, SDMA or GFR of cats with CKD, it resulted in a worsening of proteinuria [160]. For most of the NSAIDs used in cats, it is unknown if repeated long-term dosing alters the pharmacokinetics or pharmacodynamics of the drug. Since NSAIDs have historically been contraindicated in cats with CKD, no specific recommendations are available for dose adjustment in these patients. General guidelines exist on their rationale long-term use in cats, promoting the cautious adoption of the “lowest effective dose” principle when possible, leaving the frequency of dosing unchanged [7]. As not all cats are satisfactory candidates for long-term NSAIDs therapy, extreme caution is needed before making the decision to treat these patients. Cats must have a stable CKD and the maintenance of proper hydration is paramount [153].

The potential for the development of adverse reactions in dogs is well accepted, with extensive reports of toxicity over the years. Nevertheless, the strength of evidence concerning adverse drug reactions is highly variable among published studies and single NSAIDs. Although the overall incidence of side effects related to the administration of NSAIDs is currently unknown, the most serious adverse effects seem to be rather rare [68]. Acute kidney injury from NSAIDs is more likely to occur in dogs that already have a decreased renal function, probably as a consequence of the increased need for PGs to maintain an adequate RBF [69,70]. In dogs with CKD, concurrent medication that can impair these mechanisms should be considered, such as angiotensin-converting enzyme inhibitors (ACEis), angiotensin receptor blockers (ARBs) or diuretics [70,161,162].

Specific recommendations about dose adjustments of NSAIDs in dogs with CKD are not available, and general guidelines for their judicious administration are commonly observed, such as the use of the lowest effective dose and dose optimization on lean body weight, the avoidance of exposure to additional risk factors (i.e., hypotension, concurrent administration of other potentially nephrotoxic drugs) and an appropriate baseline evaluation of renal function with re-evaluation of laboratory parameters two weeks after initiating treatment [70,163]. Moreover, laboratory monitoring is recommended every 6 months in low-risk patients and every 2–4 months in at-risk patients [164]. Furthermore, observing an adequate washout period (5–7 days) is recommended for cases in which an animal is switched from one NSAID to another or has been treated with corticosteroids, to avoid adverse drug interactions [143]. In the case of long-acting corticosteroids or aspirin, a longer washout period should be considered [164].

### 6.2. Corticosteroids

Corticosteroids are widely used in veterinary and human medicine due to their potent anti-inflammatory and immunosuppressive effects.

It has been reported that corticosteroids should be avoided in the treatment of uremic dogs because they enhance protein metabolism, worsening azotemia [55]; furthermore, the administration of glucocorticoids has been associated with the onset of proteinuria in dogs without evidence of preexisting glomerular disease [71]. The mechanisms underlying glucocorticoids-induced proteinuria are poorly understood, although glomerular hypertension and ultrastructural alterations to the glomerular barrier have been implied [71,165].

Several studies have also documented that corticosteroids administration induces a rise in serum cystatin C in humans and in canine species [166]. Nevertheless, the clinical significance of these findings remains to be determined.

On the other hand, the administration of immunosuppressive therapy could be considered in dogs with persistent or progressive glomerular disease in which there is renal biopsy-supported evidence of immune-mediated pathogenesis [167]. In these cases, glucocorticoids should be limited to short-term therapy and other immunosuppressive agents should be preferred [72].

## 7. Antiproteinuric and Antihypertensive Drugs

Renin–angiotensin–aldosterone system (RAAS) inhibitors and calcium channel blockers (CCBs) are the most recommended classes of antihypertensive agents in dogs. Furthermore, RAAS inhibitors are also prescribed as first-line antihypertensive agents in CKD dogs due to their antiproteinuric effect [73]. On the other hand, CCBs and specifically amlodipine besylate have been the first choice for antihypertensive treatment in CKD cats because of their established efficacy [77,168,169]. The potential of antihypertensive agents such as RAAS inhibitors and CCBs to cause hypovolemia and hypotension and induce changes in intrarenal hemodynamics should be carefully taken into account [78]. It is thus advisable that patients receiving antiproteinuric or antihypertensive medications are clinically stable prior to the introduction of these agents, and dehydration should be avoided [72,73].

### 7.1. Renin-Angiotensin-Aldosterone System (RAAS) Inhibitors

The renin–angiotensin–aldosterone system (RAAS) is essential for the regulation of sodium balance, fluid volume and blood pressure; it is also directly implicated in the progression of hypertension, heart failure and kidney disease [170]. Angiotensin-converting enzyme inhibitors (ACEis) and ARBs selectively inhibit the RAAS and are shown to be particularly beneficial in patients with renal disease, despite a mild increase in renal function markers [73].

The reno-protective effect of ACEis has been linked to the reduction in systemic arterial pressure, glomerular capillary pressure and glomerular volume [171]; thus, they are commonly used in dogs and cats to manage hypertension and proteinuria and their use in CKD patients appears to be safe and related to positive outcomes [72,73,172,173,174,175,176].

Benazepril and its active metabolite, benazeprilat, are largely eliminated by the biliary route, with a smaller fraction being excreted in the urine; impaired renal function does not affect the clearance of this drug in dogs and cats [173,174,177,178]. In contrast, enalapril and its active metabolite, enalaprilat, are primarily eliminated by the kidney, and a 33% reduction in dose may be required in dogs with mild renal impairment [177,179]. Provided that animals are clinically stable and euvolemic before the introduction of ACEis, a severe worsening of azotemia seems to be un uncommon finding. Nevertheless, some caution is warranted when administering an ACEi to a dog or cat in stage 3 or stage 4 CKD; in these cases, a low initial starting dose (i.e., 50% of the total daily dose) followed by small incremental increases may be considered to improve renal tolerance [64,72]. In addition, a close monitoring (3–7 days after starting the therapy) of serum creatinine, potassium and systemic blood pressure should be performed to identify signs of worsening renal function, especially after any dose adjustment or the introduction of other antiproteinuric drugs [72]. Furthermore, the concurrent administration of potentially nephrotoxic drugs such as diuretics or NSAIDs should be carefully evaluated, assessing the risk and benefits [177].

Angiotensin II receptor blockers (ARBs) selectively antagonize the angiotensin II, subtype-1 (AT1) receptor, which mediates the pathologic effects of angiotensin II [74]. Telmisartan is an ARB, currently licensed in Europe for the treatment of feline proteinuria due to CKD, and was recently demonstrated to also be safe and effective for the management of systemic hypertension in feline species [180]. Telmisartan, used at antihypertensive dosages, appeared to be safe in cats with CKD of IRIS stages 1–3, with a low rate of acute exacerbation of renal azotemia noted within a 28-day study period and in a 6-month extended-use phase [74]. Telmisartan was also recently evaluated in dogs as a suitable first-line choice for the treatment of renal proteinuria, providing similar or even better results for reducing UPC in comparison with enalapril [75] and, as observed for cats, it was recently found to also be safe and effective as an antihypertensive agent in CKD dogs [76].

The potential for acute exacerbation of azotemia should be taken into account with the concurrent administration of ACEis and ARB, and careful monitoring is recommended [73].

### 7.2. Amlodipine

Amlodipine is a potent peripheral arterial dilator belonging to the class of calcium channel blockers (CCBs). These drugs directly act on vascular smooth muscle, causing a reduction in systemic vascular resistance and blood pressure with minimal cardiac effects [181].

Due to its established efficacy, amlodipine has been identified as the first-choice antihypertensive treatment in cats with hypertension and CKD [7,73,77].

The use of CCBs as monotherapy in dogs should be avoided; in fact, the local hypertensive effect of RAAS is not inhibited by CCBs, which preferentially dilate the renal afferent arteriole, potentially exposing the glomerulus to deleterious increases in glomerular capillary hydrostatic pressure. For this reason, ACEis or ARBs that preferentially dilate the renal efferent arteriole should be co-administered with a CCB in dogs [73].

### 7.3. Other Cardiovascular Drugs

Many drugs commonly used in conditions of cardiac disease, which are primarily excreted by the kidneys, may require close monitoring and dosage adjustments in animals with AKI or CKD [182].

Furosemide is a loop diuretic that decreases the reabsorption of both sodium and chloride and increases the excretion of potassium in the distal renal tubule; it is predominantly excreted by proximal tubular secretion. Furosemide is contraindicated in patients with progressive renal disease or if increases in azotemia and oliguria occur during therapy; it should be also used with caution in patients with preexisting electrolyte or water-balance abnormalities; therefore, the careful monitoring of these variables is recommended [55,60]. Nevertheless, loop diuretics may have conflicting effects on renal function in cardiorenal syndrome (CRS). By reducing renal congestion, they may improve GFR and delay the progression of CKD; on the other hand, excessive doses of diuretics may also decrease renal perfusion and, consequently, GFR. If indicated in these cases, treatment should be established with the appropriate monitoring of renal function. The combination of loop diuretics and thiazide diuretics has a synergistic effect that may cause excessive volume depletion and electrolyte disturbances; therefore, they should be used with caution in azotemic patients with CRS [78]. The concomitant administration of potentially nephrotoxic agents, such as NSAIDs, aminoglycosides and cephalosporins, should be cautiously evaluated [55].

## 8. Antithrombotic Drugs

Thromboembolism is a well-recognized complication of proteinuria in dogs and humans, although the underlying cause of hypercoagulability in dogs with protein-losing nephropathy (PLN) remains to be elucidated [183,184]. The glomerular loss of antithrombin (AT) and increased platelet aggregation secondary to hypoalbuminemia were observed in dogs with nephrotic syndrome (NS) and identified as possible causes [176,184]. While, in humans with NS, thrombosis is primarily attributed to venous thromboembolism (VTE), no strong evidence is available in dogs regarding the nature of thrombosis in PLN; nevertheless, clinical observations suggest that VTE in dogs may be more common that arterial thromboembolism (ATE) [183].

Antithrombotic therapies are recommended for clot prevention in dogs with PLN [8,185]. According to recent consensus guidelines, anticoagulants seem to be more effective than antiplatelet agents for preventing VTE in dogs [8]. Nevertheless, the mechanism of action of unfractionated heparin (UFH) and low-molecular-weight heparin (LMWH), whose target is AT, should be taken into account. Hence, the response to heparin could be highly variable depending on the AT levels and the severity of the underlying disease; in these cases, monitoring the clotting time (i.e., activated partial thromboplastin time, aPTT) is warranted [79]. Little information is available regarding the use of these drugs in dogs and cats with renal impairment but, since LMWHs are excreted by renal clearance, a prolongation of elimination time should be considered under conditions of reduced renal function [79]. In human patients with stage 4 CKD, a decreased clearance of enoxaparin and drug accumulation occur, leading to an increased half-life, drug exposure and bleeding risk; therefore, a prolongation of dosing intervals from 12 to 24 h is recommended [80].

An uncommon feature that should be considered in patients with renal disease receiving heparin, which could experience imbalances in potassium metabolism, is the heparin-induced hyperkalemia. This syndrome is related to the transient suppression of aldosterone synthesis that resolves after heparin discontinuation. It has been observed in humans receiving UFH and LMWH and also been reported in a dog with CKD [186].

Conversely to anticoagulants, the use of antiplatelet drugs such as low doses of acetylsalicylic acid and clopidogrel has been extensively described for thromboprophylaxis in dogs with PLN [41,176,183].

Acetylsalicylic acid or aspirin is an NSAID used as antiplatelet drug at low dosages, and it has been recommended for thromboprophylaxis in glomerular diseases due to its anti-inflammatory effects, which could have a role in reducing disease progression [41,176,183]; at low doses, aspirin may be more effective in inhibiting COX-1 and suppressing the production of thromboxane (TXA2) [85].

The use of low-dose aspirin in renal disease in humans with CKD rises some concerns related to the potential inhibition of renal prostaglandins, which could lead to a further deterioration in renal function [80]; moreover, several studies have shown potential risks in elderly patients and those with CKD [81,82,83,84].

On the other hand, no controlled clinical studies documenting the efficacy of low-dose aspirin are available in animals [85]. The therapeutic index of acetylsalicylic acid is narrow and, similarly to other NSAIDs, caution should be used under conditions of renal hypoperfusion [86]. According to these considerations, the concomitant administration of other NSAIDs should be avoided in these patients and renal function should be monitored.

The prevalence of PLN seems to be lower in cats and, conversely to dogs, there is no evidence supporting the adoption of antithrombotic medications [8,185,187]. Although it is not licensed for use in cats and its efficacy remains unproven, acetylsalicylic acid at low doses has been encountered as antiplatelet agent in cats for the prevention of thrombosis and in particular ATE; reported dosages range widely (i.e., 5–80 mg per cat every 72 h), and special attention should be paid to the prolonged time of excretion in feline species [85,86]. Since clopidogrel was shown to be more effective than aspirin in reducing the likelihood of recurrent cardiogenic ATE [188], recent consensus guidelines on the rational use of antithrombotic recommend using clopidogrel rather than aspirin in cats when required [8].

Clopidogrel is a platelet inhibitor belonging to the class of thienopyridines that inhibits diphosphate (ADP)-receptor-mediated platelet activity [85]. The pharmacokinetics of clopidogrel is influenced by genetic polymorphisms in the genes encoding for cytochrome (CYP) 2C19, determining a high inter-individual variability in treatment response [89]. Although dose adjustment seems to be unnecessary under conditions of reduced renal function, therapeutic experience with clopidogrel is limited in human patients with advanced CKD [80,87,88]. Concerns exist regarding the benefits deriving from the use of clopidogrel in CKD patients and, specifically, in end-stage kidney disease (ESKD). Hence, a lower antiplatelet effect of clopidogrel was observed in CKD patients [90] and has been associated with an increased risk of death, death from bleeding, and hospitalization for bleeding in patients with ESKD [88]; as a result, the summary of product characteristics of clopidogrel recommends caution when using clopidogrel in the CKD population [80]. Clinical studies on clopidogrel in dogs and cats with CKD are lacking; according to a recent study, no differences in clopidogrel metabolite concentrations were found between healthy dogs and those with PLN and early CKD stages, suggesting that the drug’s metabolism is not altered in these patients [91]. Nevertheless, caution should be adopted when administering clopidogrel in dogs and cats with advanced CKD.

Although a combined administration of clopidogrel and aspirin can be used, due to their different mechanisms of action, an increased risk of gastrointestinal side effects has been reported in humans and is also likely to occur in animals [189].

## 9. Gastroprotectants, Antiemetics and Appetite Stimulants

The gastrointestinal complications of CKD, including nausea, vomiting, uremic stomatitis and gastrointestinal erosions, are common in dogs and cats with advanced renal disease. The treatment of these complications is largely symptomatic and include the administration of specific drugs to limit gastric acidity, suppress nausea and vomiting and provide mucosal protection [1].

Oral antacids comprise a group of inorganic and relatively insoluble salts of aluminum hydroxide, calcium carbonate and magnesium hydroxide that lack systemic effects [190]. In renal failure, magnesium and aluminum accumulation may be a problem and aluminum toxicity has been documented in dogs with advanced renal failure [92,191]. Further concerns related to these drugs arise from their interference with the absorption of concurrent orally administered medications [40].

Histamine type-2 receptor antagonists (H_2_RAs) include cimetidine, ranitidine and famotidine, which exert their action through the inhibition of acid secretion by competitively blocking H_2_ receptors on the gastric parietal cells. Dose adjustments of H_2_RAs based on GFR are recommended in humans because of the renal elimination of these drugs. Mental status changes and other central nervous system disturbances disturbances have been reported in people with decreased GFR when receiving H_2_ without appropriate dose reductions [93]. Although no specific indications are available in dogs and cats [94], either a dose reduction or extended dosing interval can be used at IRIS Stages 3 and 4.

Omeprazole, pantoprazole, esomeprazole and lansoprazole are classified as proton-pump inhibitors (PPIs) and are identified as first-choice treatments of acid-related gastrointestinal ulceration and erosion. Retrospective reports have linked PPIs to acute interstitial nephritis, AKI and CKD in people but the quality of evidence underlying this association, as well as for other adverse effects, is consistently low in dogs and cats [94,95,96,192,193]. To date, there is no evidence supporting the prophylactic use of gastroprotectants in dogs and cats with CKD IRIS stage 1–3 and additional studies are warranted to determine the benefits of acid suppression in animals with IRIS stage 4 disease; no dose adjustment is required in patients with renal impairment [94].

Sucralfate is a complex salt of sucrose octasulfate and aluminum hydroxide that provide protection of the damaged mucosa through the formation of stable complexes with proteins in an acidic environment [194]. Although sucralfate is a relatively safe compound with minimal adverse effects, caution should be used with long-term treatment in patients with renal insufficiency to avoid aluminum intoxication; moreover, the possible influence on the absorption of other orally administrated drugs should be considered [195,196].

Metoclopramide is a dopamine receptor antagonist, widely used in dogs and cats, to stimulate the stomach and upper-small-intestine motility. This action helps prevent esophageal reflux and move *ingesta* from the stomach into the intestine. Metoclopramide is also used in dogs and, less commonly, in cats to treat nausea [60]. The use of this drug at standard constant rate infusion dosages (1–2 mg/kg/day) may cause tremor and ataxia in azotemic patients. The dose could be reduced by up to the 25–50% of the standard dose and titrate to a dosage that elicits a therapeutic effect without tremor [64].

Maropitant is a selective neurokinin-1 (NK-1) receptor antagonist whose mechanism of action is to effectively block the binding of emetic-eliciting substance P, the most potent tachykinin, at the NK-1 receptor. It is highly bound to plasma proteins in dogs and cats, and is mainly excreted by the liver; thus, it is recommended to use it with caution with other highly protein-bound medications and in patients with hepatic dysfunction. Maropitant is safe and effective in the treatment of vomiting induced by several conditions in dogs, including motion sickness, and preventing vomiting induced by chemotherapy [97,98,197,198]; it was also shown to be safe and effective in cats and daily administration orally for 2 weeks resulted in a statistically significant decrease in vomiting in cats with CKD [99,199].

Ondansetron is a 5HT_3_ receptor antagonist used for severe vomiting in dogs and cats. No canine data are available for ondansetron pharmacokinetics, while, in cats, subcutaneous bioavailability was found to be prolonged (3.2 h) in comparison with oral and intravenous routes [200]. Ondansetron is extensively metabolized by the liver. The pharmacokinetics of ondansetron was evaluated in healthy geriatric cats, cats with CKD and liver disease, finding no significant differences between healthy geriatric cats and those with renal disease [100]. Thus, renal dysfunction seems unlikely to affect the pharmacokinetics of ondansetron in these patients [100].

### Mirtazapine

Mirtazapine is used as oral or transdermal formulation for nutritional support in both dogs and cats with acute and chronic illness.

The mechanisms of action include antagonism of the serotonin 2C (5HT2C) and histamine 1 (H1) receptors, both of which are involved in appetite regulation, and an antiemetic action through the inhibition of serotonin 3 receptor (5HT3) [103,201,202,203]. Due to its high affinity for 5HT3, competition with 5HT3-receptor antagonists (e.g., ondansetron) should be considered when choosing antiemetic and anti-nausea regimens.

The efficacy of mirtazapine as an appetite stimulant has been thoroughly studied in cats. In feline patients with CKD, it provides significative appetite stimulation and weight gain [101,103,201,204,205,206]. The half-life of oral mirtazapine is prolonged in elderly cats (12.1 h) and cats with CKD (15.2 h) and/or liver disease (15.1 h) [101,207]. There are differences in pharmacokinetics among formulations, with a lower mean peak and total plasma concentration recorded with a transdermal ointment than oral dosing [208]. The most commonly reported adverse effects include neurologic signs, agitation, vocalization, hypersalivation, tachypnea, tachycardia and lethargy, and were more commonly reported in cats receiving 3.75 mg than in cats receiving 1.88 mg; therefore, the lower dosage seems to be more appropriate as a starting dose [102,103]. Given the prolonged half-life of mirtazapine in CKD cats, oral administration of the medication every 48 h is recommended [101]. To minimize its adverse effects, the dosage should be titrated to the lowest effective dose for appetite stimulation with both the oral and transdermal formulations. The recommendations provided by the manufacturer of a transdermal formulation (Mirataz 20 mg/g, Dechra Veterinary Products^®^) state that the product has not been studied in patients with severe CKD and for a treatment duration of longer than 14 days; nevertheless, a good safety and efficacy profile was recently shown in a study evaluating the clinical efficacy of mirtazapine in cats with stage 2 and 3 CKD, receiving a compounded transdermal formulation for 21 days [206]. Side effects reported by the manufacturer include vomiting, polyuria and an increase in BUN consequent to dehydration.

Fewer studies regarding the use of mirtazapine have been conducted in dogs, with limited evidence supporting it as an appetizing agent in comparison with feline species. The suggested dose interval for dogs is 0.6–1 mg/kg every 12 h, but studies evaluating pharmacokinetic properties in dogs with renal disease have not been conducted to date [104].

## 10. Antileishmanial Drugs

Canine leishmaniasis is a common cause of glomerulopathy (particularly glomerulonephritis), which can cause proteinuria and progressive chronic kidney disease [113,209,210,211,212]. When leishmaniosis-induced kidney disease is identified, together with an active form of the disease, specific treatment should be started immediately, either alone or together with the standard treatment for CKD. Nevertheless, treatment should be tailored to each individual case and the risk-to-benefit ratio should be carefully considered [113,213].

The association of meglumine antimoniate and allopurinol is the most widely described and effective treatment for leishmaniosis; when this regimen is not feasible, the alternative combination of miltefosine and allopurinol could be administered [214,215]. All these drugs may improve the patient’s clinical condition and prevent the progression of kidney disease [106,216,217,218]. Although nephrotoxicity is a recognized side effect of antimonials, no impact on kidney function was observed in dogs treated with meglumine antimoniate and the only reported evidence of pathological renal damage (mainly tubular lesions) was not associated with clinical or clinicopathological alterations [105,106,107]. Nevertheless, the manufacturer of a veterinary formulation (Glucantime, Boehringer Ingelheim^®^ Ingelheim, Germany) recommends reducing dosage in the case of renal impairment and monitoring serum creatinine and proteinuria during treatment; administration in cases of severe renal insufficiency is discouraged.

Miltefosine is generally considered a second-line treatment for canine leishmaniosis; it shows a good safety profile and seems to have a low impact on renal function. The only reported adverse effects are transient gastrointestinal signs such as vomiting and diarrhea.

Allopurinol is a purine analogue leishmaniostatic agent used to treat canine leishmaniosis over long periods to maintain a low parasite load. Xanthine urolithiasis, which can potentially result in kidney mineralization, was observed with a prolonged therapy [113,219]. Given xanthine lithiasis’ potential impact on kidney function (particularly when the upper urinary tract is involved), a closer follow-up through urinalysis and ultrasound is required to detect the early onset of such an adverse effect. If xanthine uroliths were detected or suspected, the dose of allopurinol can be reduced to 10 mg/kg once a day or less [113,213,214] and other approaches to minimize xanthine-associated crystalluria can be started (e.g., increase in water intake, specific purine-restricted diets, urine alkalinization).

Many dogs with leishmaniosis may also require antiproteinuric treatment, which should be based on the available guidelines, considering the previously discussed remarks on renal impairment [214,220].

Leishmaniasis in cats is a possible underestimated cause of renal disease. According to the limited information available on treatment regimens, which is mostly based on single case reports, the same protocols that are used in dogs are generally prescribed in cats [221,222]. However, it should be specified that the efficacy and safety of these protocols have never been evaluated in feline-controlled studies and cats, particularly those affected by renal disease, should be monitored very carefully for adverse effects during treatment. The administration of allopurinol was found to be associated with the onset of acute of kidney injury in cats [114], while no nephrotoxicity was recorded for meglumine antimoniate or miltefosine [223].

## 11. Conclusions

**Figure 1:**
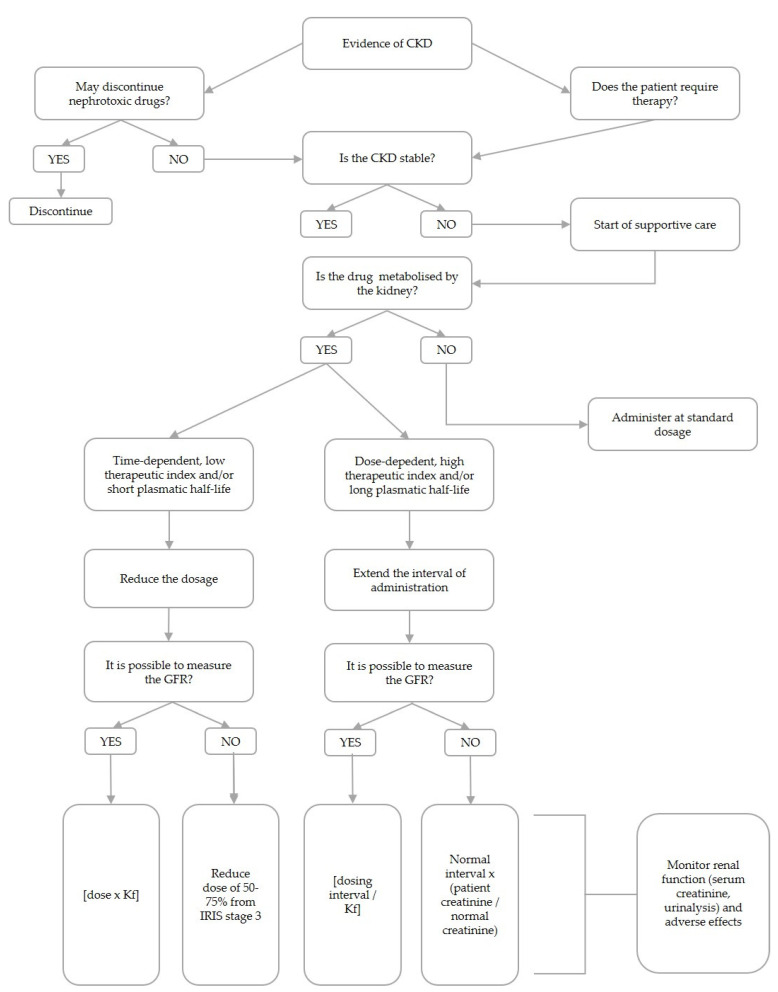
Proposed practical guideline for drug dosage adjustments in canine and feline chronic kidney disease. CKD: Chronic Kidney Disease; GFR: Glomerular Filtration Rate; Kf: Filtration Coefficient.

Practical guidelines on dosage adjustment regimens seem to be lacking in the veterinary literature and most of the available indications are based on the measurement of GFR by means of plasma clearance methods. In contrast to humans, the assessment of GFR in dogs and cats is generally not performed in clinical practice for both financial and practical reasons and, to date, it is mainly performed in research settings. Typically, dose adjustments in humans are made when GFR drops to approximatively 0.7 mL/kg/min, roughly equivalent to a serum creatinine of 3.5 mg/dL in cats. On the other hand, data in dogs are still lacking and equivalent dose adjustment studies are warranted in both species. For these reasons, the recommendations for dosage adjustment based on indirect tests evaluating kidney function and reflecting the severity of the CKD can be extrapolated from human studies and adapted from expert opinion. Practical dose adjustments of drugs characterized by a high therapeutic index should be taken into account from IRIS stage 3, while drugs with a narrow therapeutic window could require dosage adjustments at an earlier stage (i.e., IRIS stages 2) (Figure 1). Moreover, given the lack of safety studies in CKD populations, specific recommendations regarding dosage adjustment are seldom provided by manufacturers. As a result, potentially nephrotoxic drugs might not be prescribed in patients with renal impairment even if they are clearly required, with a possible negative impact on treatment outcomes and quality of life.

In conclusion, treatment decisions should always be made on a case-by-case basis, assessing risks and benefits; the severity of the disease and the effective need for a given medication should be carefully evaluated. Furthermore, indications provided by the manufacturers should be integrated and compared with the available literature to obtain a more complete overview of the decision-making process.

From a future perspective, prospective clinical trials evaluating the efficacy and safety of specific medications in dogs and cats with various stages of CKD could help to provide practical guidelines for dosage adjustment in veterinary medicine.

## Figures and Tables

**Table 1:** Summary of the main recommendation on the use of the revised class of drugs in patients with impaired renal function. CKD: chronic kidney disease; SDD: single daily dose; TDM: therapeutic drug monitoring; GFR: glomerular filtration rate; NSAIDs: non-steroidal anti-inflammatory drugs; ACEIs: Angiotensin II Converting Enzyme Inhibitors; ARBs: angiotensin receptor blockers; UPC: urinary protein to creatinine ratio; SBP: sistolic blood pressure; CCBs: calcium channel blockers; CRS: cardiorenal syndrome; LMWHs: low-molecular-weight heparins; H_2_RAs: histamine type-2 receptor antagonists; IRIS: International Renal Interest Society; PPis: proton pump inhibitors; AKI: acute kidney injury.

Class	Drug	Comments
Antimicrobial agents	Aminoglycosides	Potentially nephrotoxic. Their use is generally discouraged in CKD patients [55]. When clinically indicated, the patients should be hemodynamically stable, and the concomitant use of other nephrotoxic drugs should be avoided. SDD regimen is recommended in association with TDM. The duration of treatment should be minimized (<7 days) [59].
	Penicillins	High therapeutic index [60]. Due to the possible accumulation, dose adjustment could be considered for ampicillin in dogs with severe azotemia [61]. Although penicillins have been classified as “probably safe” in cats with CKD [7,13], the need for dose adjustment in azotemic feline patients remains to be determined [62].
	Cephalosporins	Dosage regimen adjustment based on an extension of the administration interval is recommended in humans with a moderate to severe reduction in GFR [63]; interval extension should be considered in dogs and cats with moderate or severe CKD [64].
	Sulfonamides	Potential nephrotoxicity due to crystalluria and hypersensitivity reactions. Dehydration should be avoided [65]. In patients with reduced renal function dose adjustment could be considered (i.e., halving the dose or doubling the interval of administration) [64].
	Tetracyclines	Increased risk of nephrotoxicity for water-soluble medications (e.g., oxytetracycline). In patients with altered renal function, doxycycline or minocycline should be preferred [60].
	Fluoroquinolones	Undergo renal and non-renal elimination pathways. Dose adjustment may not be required in dogs [66]. Due to the high risk of retinopathy, an interval extension is recommended in CKD feline patients with moderate disease [7].
Anti-inflammatory drugs	NSAIDs	Potentially nephrotoxic [67,68]. When clinically indicated, the patients should be hemodynamically stable and the concomitant use of other nephrotoxic drugs should be avoided; close monitoring of renal function and administration of the lowest effective dose are recommended [7,69,70]. A washout period should be envisaged between different NSAIDs molecules and corticosteroids [69].
	Corticosteroids	Poor evidence of nephrotoxicity in dogs and cats. Possible worsening of azotemia and development of proteinuria reported in dogs [71].
Antiproteinuric and antihypertensive drugs	ACEis	Potentially nephrotoxic. Patients should be hemodynamically stabilized before administration and adoption of a lower starting dose and monitoring of renal function, SBP and serum electrolytes are recommended [72]. Caution should be used in an advanced stage of CKD and if administered in association with ARBs [73].
	ARBs	Potentially nephrotoxic. Telmisartan is safe and effective in reducing UPC and SBP in dogs and cats with CKD [74,75,76]. Caution should be used in advanced CKD stages and if administered in association with an ACEi [73].
	CCBs	Amlodipine is a safe antihypertensive agent in cats with CKD [7,73,77]. Since CCBs preferentially dilate the renal afferent arteriole, exposing the glomerulus to increased hydrostatic pressure, their use as the sole antihypertensive drug in dogs is discouraged [73].
Diuretics	Furosemide	Potentially nephrotoxic due to excessive volume depletion. Contraindicated in patients with unstable renal function or volume depletion conditions; close monitoring of renal function is strongly recommended and association with other nephrotoxic drugs is discouraged [55,60]. Its use may be required in CRS [78].
Antithrombotic agents	LMWHs	Risk of accumulation should be considered under conditions of reduced renal function [79]. Interval extension is recommended in humans with stage 4 CKD [80].
	Acetylsalicylic acid	Potentially dangerous in human CKD patients [81,82,83,84].No controlled clinical studies are available in animals [85]. Caution should be used under renal hypoperfusion conditions [86]. The concomitant administration of other NSAIDs should be avoided and renal function should be monitored. Prolonged time of excretion in cats [85].
	Clopidogrel	Dose adjustments seem to not be required in humans with CKD [87,88]; however, the marked variability in pharmacokinetics of clopidogrel justifies its cautious use in patients with advanced CKD [80,88,89]; a lower antiplatelet effect has been observed in CKD patients [90]. Drug metabolism may not be altered in dogs with PLN and early CKD [91].
Gastroprotectants	Antacids	Although they generally lack systemic effects, the possible accumulation of aluminum and hypermagnesemia should be considered for aluminum/magnesium-containing salts in dogs with advanced renal failure [92]. No specific indications for dose adjustment are available in dogs and cats with CKD.
	H_2_RAs	Dose adjustments recommended in humans [93]. Although no specific indications are available in dogs and cats [94], either decreasing the dose or extending the dosing interval can be used in IRIS Stages 3 and 4.
	PPIs	Nephrotoxicity (interstitial nephritis) reported in humans [95,96]. Poor evidence of nephrotoxicity in dogs and cats [94].
	Sucralfate	Relatively safe compound. Caution should be used with long-term treatment in patients with renal insufficiency to avoid aluminum intoxication [94].
Antiemetics	Metoclopramide	Administration at standard constant rate infusion dosages (1–2 mg/kg/day) may cause tremors and ataxia in azotemic patients. The dose could be reduced by up to the 25–50% of the standard dose and titrate to dosage, which elicit a therapeutic effect without tremor [64].
	Maropitant	Safe in dogs and cats with renal impairment [97,98,99].
	Ondansentron	Safe in cats with renal impairment [100]. No pharmacokinetic data are available for dogs.
Appetite stimulants	Mirtazapine	1.88 mg/cat q 24–48 h may be suitable as a starting dose in cats with CKD [101,102,103]. Limited data are available for dogs [104].
Antileishmanial drugs	Meglumine antimoniate	Despite the limited evidence of nephrotoxicity, dose reduction and a monitoring of renal function are recommended [105,106,107]. Use in dogs with advanced renal failure is discouraged. Limited data on use in cats.
	Miltefosine	Low impact on renal function in dogs [105,108,109,110,111,112]. Limited data on use in cats.
	Allopurinol	In cases of xantinuria, mineralization or uroliths, the interval of administration should be prolonged (i.e., 10 mg/kg q24 h) [113]. Limited data are available for cats; development of AKI reported [114].

## Data Availability

Not applicable.
